# Early vascular parameters in the micro- and macrocirculation in type 2 diabetes

**DOI:** 10.1186/s12933-018-0770-4

**Published:** 2018-09-19

**Authors:** Dennis Kannenkeril, Agnes Bosch, Joanna Harazny, Marina Karg, Susanne Jung, Christian Ott, Roland E. Schmieder

**Affiliations:** 1 Department of Nephrology and Hypertension, University Hospital of the University of Erlangen-Nuremberg, Ulmenweg 18, 91054 Erlangen, Germany; 2 Department of Pathophysiology, University of Warmia and Mazury, Olsztyn, Poland; 3 Department of Cardiology, University Hospital of the University of Erlangen-Nuremberg, Erlangen, Germany

**Keywords:** Inter capillary distance, Central pulse pressure, Diabetes mellitus type 2, Micro-and macrocirculation

## Abstract

**Background:**

Diabetes converts from a metabolic disorder into a predominantly vascular disease, once its duration extends over several years or/and when additional cardiovascular risk factors such as hypertension coexist. In a cross-sectional analysis we analyzed various vascular parameters in the renal, retinal and systemic circulation, with the goal to identify which vascular parameter of early organ damage is the earliest that can be clinically detected.

**Methods:**

In 111 patients with type 2 diabetes (T2DM) (off any anti-diabetic medication for at least 4 weeks) and 54 subjects without T2DM we compared various parameters of early vascular remodeling in the same patient: urinary albumin creatinine ratio ([UACR], early morning spot urine) and estimated glomerular filtration rate (eGFR), retinal capillary flow (RCF) and intercapillary distance (ICD) as parameters of capillary rarefaction, wall-to-lumen ratio (WLR) of the retinal arterioles [all assessed by Scanning Laser Doppler Flowmetry], and central systolic pressure (cSBP) and central pulse pressure (cPP) [measured by pulse wave analysis, Syphygmocor] both reflecting vascular stiffness of large arteries.

**Results:**

Compared to subjects without T2DM, patients with T2DM (diabetes duration: median 48 months, interquartile range 24–88 months) were older (59.8 ± 7.3 vs 43.4 ± 12.9 years, p < 0.001), more females (33.3 vs 20.4%, p < 0.001), but 24-h systolic and diastolic blood pressure did not differ between the two groups. The analysis adjusted for age, gender and cardiovascular risk factors revealed that ICD (23.9 ± 5.1 vs 20.8 ± 3.5 µm, p value = 0.001) and cPP (41.8 ± 11.7 vs 34.8 ± 10.6 mmHg, p value < 0.001) were significantly higher and eGFR (91.7 ± 9.9 vs 95.9 ± 17.3 ml/min/1.73 m^2^, p value < 0.001) was significantly lower in patients with T2DM than in subjects without T2DM.

**Conclusion:**

These data suggest that at similar blood pressure capillary rarefaction in the retinal circulation (ICD), decreased eGFR in the renal circulation and increased central pulse pressure (cPP) of large arteries are earlier detectable than other vascular remodeling parameters of the micro- (WLR, RCF, UACR) and macrocirculation (cSBP) in patients with T2DM.

*Trial registration* Trial registration number: NCT02471963, Date of registration: June 15, 2015, retrospectively registered; Trial registration number: NCT01319357, Date of registration: March 21, 2011, retrospectively registered; Trial registration number: NCT02383238, Date of registration: March 9, 2015, retrospectively registered; Trial registration number: NCT00152698, Date of registration: September 9, 2005, prospectively registered; Trial registration number: NCT00136188, Date of registration: August 26, 2005, prospectively registered

## Background

The role of type 2 diabetes (T2DM) in the development of micro- and macrovascular complications of T2DM like nephropathy, retinopathy, coronary, cerebrovascular and peripheral artery disease has been well described. Numerous pathogenic mechanisms like endothelial dysfunction, inflammations and various metabolic factors beyond glucose levels contribute to these vascular complications in patients with T2DM [1, 2]. The National Health and Nutrition Examination Survey (NHANES 1999–2004) showed that an estimated three out of five people (57.9%) with diagnosed diabetes have one or more vascular complications. The prevalence of coronary heart disease, retinopathy and nephropathy was 9.1%, 18.9% and 27.8% respectively [3].

Early detection of vascular remodeling and functional impairment in patients with T2DM is important to avoid the progression of vascular damage and ultimately irreversible complications. A close association between micro- and macrovascular complications of T2DM has been described [4]. Even a mild stage of diabetic retinopathy is associated with higher risk of coronary heart disease and stroke independent of traditional risk factors [5–7]. The presence of any degree of diabetic retinopathy is also linked with increased risk of all-cause mortality [8].

Likewise, the presence of microalbuminuria or reduced estimated glomerular filtration rate (eGFR), markers of diabetic nephropathy, in patients with T2DM are associated with twofold increase in risk for cardiovascular events, including cardiovascular death [9–11]. In the ADVANCE study T2DM patients with both albuminuria and reduced eGFR had more than threefold higher risk for cardiovascular events (cardiovascular death, nonfatal myocardial infarction, or nonfatal stroke) [12]. Both albuminuria and reduced eGFR together also amplify the risk of progression to end-stage-renal-disease [13]. The predictive value of microalbuminuria for the progression of kidney damage in patients with T2DM is high, which was confirmed several years ago [14]. In the UK Prospective Diabetes Study (UKPDS) around 2% per year of the diabetic patients progressed from normo- to micro-albuminuria and a further 2% from microalbuminuria to clinical grade proteinuria [15].

Furthermore, Identification of microvascular changes overall allows us to judge the development of the risk in patients. Changes in albuminuria have been found to predict mortality and cardiovascular and renal outcome in the ONTARGET trial [16].

In a cross-sectional analysis we analyzed various parameters of the renal, retinal and systemic circulation in parallel in the same patient, with the goal to identify the earliest vascular parameters of organ damage, which can be clinically detected in T2DM patients in early stage of their disease.

## Methods

### Study design

This is a cross-sectional analysis of baseline data of various interventional studies in subjects with and without T2DM, all performed at the Clinical Research Unit of the Department of Nephrology and Hypertension, University of Erlangen-Nuremberg, Germany [NCT02471963, NCT01319357, NCT02383238, NCT00152698 and NCT00136188]. Participants were recruited by advertising in local news-papers in the area of Erlangen-Nuremberg, Germany, and eligible participants were enrolled consecutively. Written informed consent was obtained before study inclusion. The protocol of each study was approved by the local ethics committee (University of Erlangen-Nuremberg), and the studies were conducted in accordance with the Declaration of Helsinki and the principles of good clinical practice guidelines. None of the financial sponsors did contribute to data collection, interpretation of the data, or the decision to approve and submit the manuscript. Any adverse events that occurred during the study were recorded.

### Study population

A total of 111 Caucasian men and women with T2DM (defined by fasting glucose ≥ 126 mg/dl or HbA1c ≥ 6.5% or on blood glucose lowering medication) [NCT02471963, NCT01319357, NCT02383238] and 54 without T2DM [NCT00152698, NCT00136188] were included. Subjects with T2DM were off any anti-diabetic medication for at least 4 weeks before vascular assessment were obtained.

Subjects with T2DM were in the early stage of their disease, because they presented without irreversible end-organ-damage. Individuals were excluded if they had any other form of diabetes were being treated with insulin or when the oral anti-diabetic drug could not be withdrawn, had a HbA1c level ≥ 10% (86 mmol/mol), had a fasting plasma glucose (FPG) level > 240 mg/dl, had blood pressure (BP) ≥ 180/110 mmHg, had an eGFR < 60 ml/min/1.73 m^2^, or had a body mass index (BMI) > 40 kg/m^2^ or suffered from cataract or glaucoma or history of epilepsy, myocardial infarction, unstable angina pectoris, percutaneous coronary intervention, or heart failure within the previous 6 months. The results were compared to a group of 54 male and female Caucasian individuals without T2DM, who were recruited in parallel and had the same clinical work-up as patients with T2DM. Non-T2DM-subjects with any significant disease were excluded.

### Clinical parameters

Demographic data were recorded at the first visit of the study and a fasting blood sample was taken in order to measure HbA1c, FPG, lipid levels, and other biochemical parameters (e.g. creatinine, liver enzymes). Office BP and heart rate measurements were taken in a seated position after 5 min of rest performed in a standardized fashion according to guideline recommendations [17]. Office BP was measured three times and the mean value of these three measurements was calculated and defined as the trough mean sitting BP. Twenty-four hour ambulatory BP was measured in parallel with Spacelab 90207 (Spacelabs Health Care, WA, USA). Measurements were taken every 15 min throughout the day and every 30 min during the night.

### Vascular assessment

We assessed various microvascular parameters of retinal circulation [intercapillary distance (ICD), retinal capillary flow (RCF) and wall-to-lumen ratio (WLR)] and of renal circulation [estimated glomerular filtration rate (eGFR) and urinary albumin creatinine ratio (UACR)] as well as various macrovascular parameters of systemic circulation [central systolic pressure (cSBP) and central pulse pressure (cPP)], both reflecting vascular stiffness of large arteries, with the goal to identify which vascular parameter of early organ damage is the earliest that can be clinically detected.

### Macrovascular parameters

Macrovascular parameters were evaluated through pulse wave analysis using the SphygmoCor™ system (AtCor Medical, Sydney, Australia). The obtained central arterial waveforms were used to calculate cSBP and cPP [18, 19].

### Retinal vascular parameters

Scanning Laser Doppler Flowmetry (SLDF) at 670 nm was performed using a Heidelberg retina flowmeter (Heidelberg Engineering, Germany). The perfusion images were analyzed by automatic full field perfusion image analysis (AFFPIA, SLDF version 4.0) [20, 21]. This program was used to measure microvascular parameters, including RCF, WLR and ICD in a standard manner as described in former studies [22, 23].

### Renal parameters

eGFR was calculated using Chronic Kidney Disease Epidemiology Collaboration (CKD-EPI) equation [24, 25]. Early morning spot urine was collected to calculate UACR, as previously suggested [26].

### Statistic

Primary objective was to identify which vascular parameter of early organ damage can be clinically detected in subjects with T2DM compared to subjects without T2DM. Normal distribution of data was confirmed by Kolmogorov–Smirnov test before further analysis. Normally distributed data were compared by unpaired Student t tests. Data are given as mean ± standard deviation. For non-normally distributed parameters, Mann–Whitney-U-Test was used for further analysis. Two-tailed values of p < 0.05 were considered statistically significant.

Covariance analyses were performed using univariate linear analysis. Adjustment for model 1 included age, gender and for model 2 age, gender, BMI, FPG, HbA1c and HDL-cholesterol. Since the blood pressure was similar between the groups, the results were not adjusted for blood pressure. Non-adjusted and adjusted p-values are given in the text of the result and in the tables. Box plots (median, interquartile ranges) are used to illustrate our results. All analyses were performed using IBM SPSS Statistics 21.0.0.2, USA.

## Results

### Study population

The clinical characteristics of the study population consisted of 111 subjects with T2DM and 54 subjects without T2DM (Table 1). Median of diabetes duration was 48 months (interquartile range 24–88 months). Compared to subjects without T2DM, subjects with T2DM were older and more frequently women. Mean HbA1c of subjects with T2DM was 6.8%, with a range of 5.4–9%. Sixty-four subjects with T2DM had coexisting arterial hypertension and 62 out of 64 were on antihypertensive therapy. Of note, 24-h, ambulatory and office systolic and diastolic BP did not differ between the two groups. Arterial hypertension was well controlled in subjects with T2DM, who were treated with an average of 1.8 ± 0.9 antihypertensive medications.

**Table 1:** Clinical characteristics of study population

Clinical characteristics	Non-T2DM-subjects (n = 54)	T2DM-subjects (n = 111)	*p* value
Age (years)	43.4 ± 12.9	59.8 ± 7.3	< 0.001
Sex (m/f)	43/11	74/37	< 0.001
BMI (kg/m^2^)	27.4 ± 4.7	29.9 ± 4.4	0.001
Systolic office BP (mmHg)	129.4 ± 13.9	129.2 ± 13.6	0.93
Diastolic office BP (mmHg)	78.8 ± 9.0	79.2 ± 8.6	0.80
HR (bpm)	69.7 ± 9.6	68.9 ± 9.7	0.61
Systolic 24 h ambulatory BP (mmHg)	130.4 ± 10.8	129.3 ± 11.4	0.72
Diastolic 24 h ambulatory BP (mmHg)	77.4 ± 5.6	78.9 ± 8.3	0.49
Fasting plasma glucose (mg/dl)	86.8	136.2	< 0.001
HBA1c (%)	5.6 ± 0.3	6.8 ± 0.8	< 0.001
eGFR (ml/min/1.73 m^2^)	95.9 ± 17.3	91.7 ± 9.9	0.10
Serum cholesterol (mg/dl)	211.8 ± 43	205.4 ± 37.2	0.33
Serum LDL-cholesterol (mg/dl)	133.6 ± 31.9	141.5 ± 29.7	0.12
Serum HDL-cholesterol (mg/dl)	57.9 ± 13.9	47.1 ± 9.9	< 0.001

Data are given as mean ± SD

*BMI* body mass index, *BP* blood pressure, *LDL* low density lipids, *HDL* high density lipids, *eGFR* estimated glomerular filtration rate, *HR* heart rate

### Microvascular parameters

**Fig. 1:**
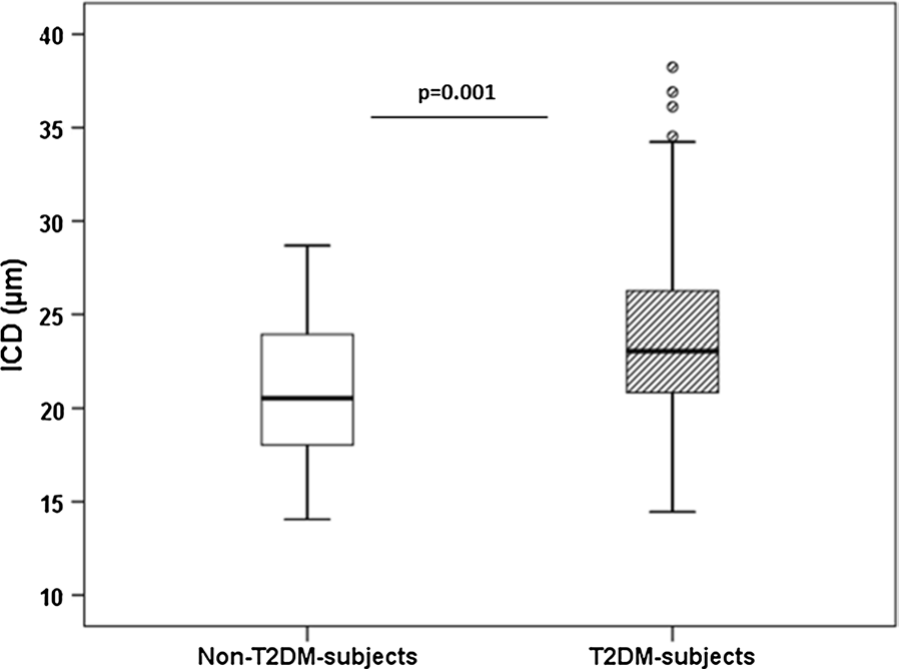
ICD in non-T2DM- and T2DM-subjects

In subjects with T2DM we observed significantly higher ICD of retinal capillaries compared to subjects without T2DM (20.8 ± 3.5 vs 23.9 ± 5.1 µm, p value < 0.001). When potential confounders such as age and gender were entered in the covariance analysis (model 1), ICD was still found to be elevated in subjects with T2DM (adjusted p value < 0.001). ICD was also elevated in subjects with T2DM after adjustment for age, gender and cardiovascular risk factors (model 2) (adjusted p value = 0.001) (Fig. 1). Other retinal vascular parameters such as WLR and RCF, were not significantly different between the groups after adjustment in model 1 and model 2 (all ns).

**Fig. 2:**
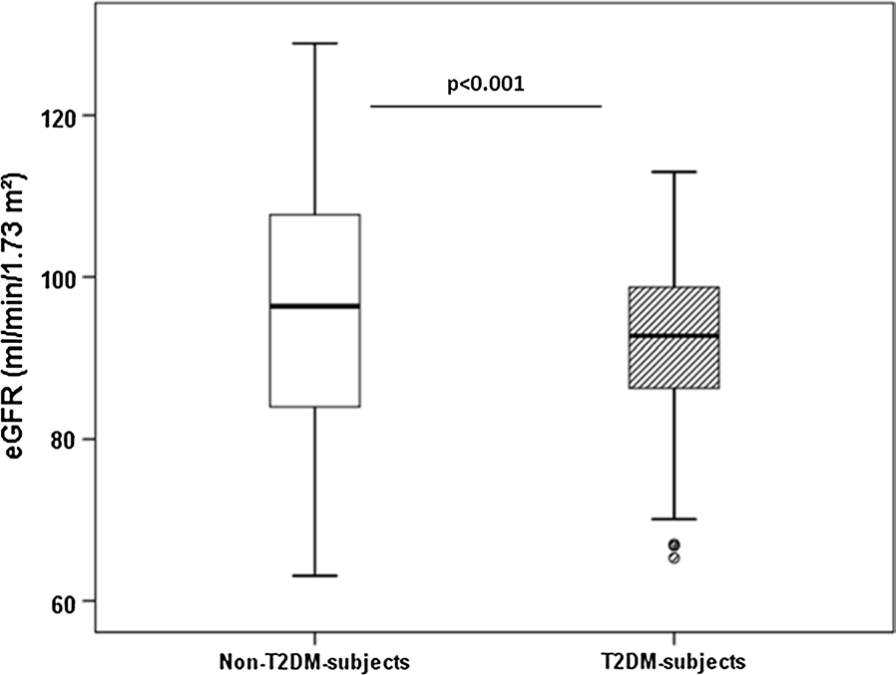
eGFR in non-T2DM- and T2DM-subjects

We did not observe any difference in UACR between two groups, after adjusting for potential confounders. eGFR was significantly lower in the patients with T2DM compared to the subjects without T2DM (91.7 ± 9.9 vs 95.9 ± 17.3 ml/min/1.73 m^2^, adjusted p values for model 1 and 2 are < 0.001) (Fig. 2). Diabetic disease duration, FPG and HBA1c were not correlated with ICD and eGFR, respectively.

### Macrovascular parameters

**Fig. 3:**
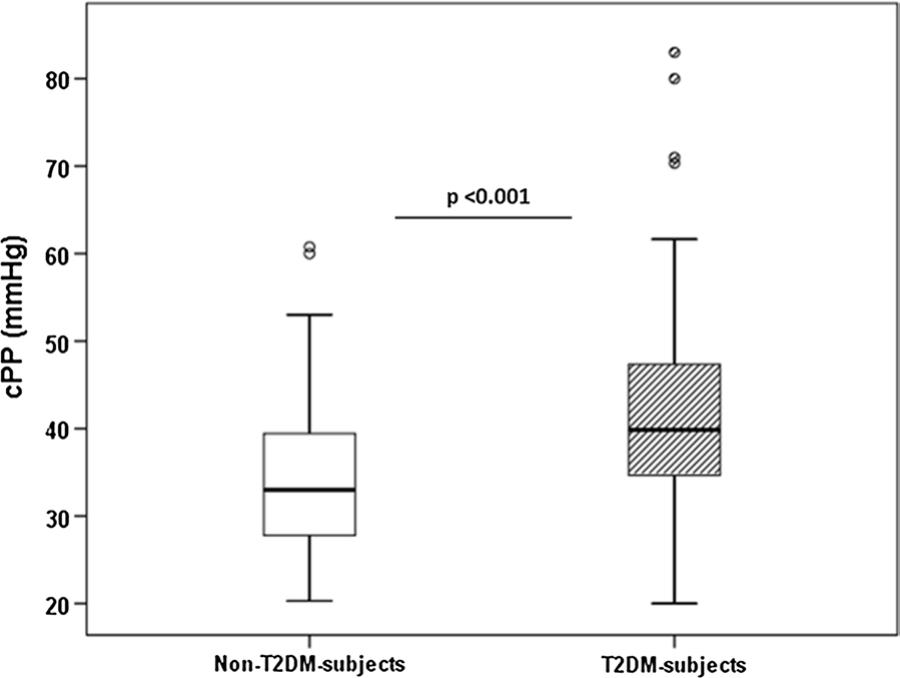
cPP in non-T2DM- and T2DM-subjects

We found significantly higher cPP of large arteries in patients with T2DM compared to subjects without T2DM (41.8 ± 11.7 vs 34.8 ± 10.6 mmHg, p value = 0.001). Covariance analysis with model 1 changed the result to an insignificant result (adjusted p value = 0.31), however after adjusting for all potential confounders (model 2) the different cPP between the 2 groups remained significant (adjusted p value < 0.001) (Fig. 3). Another vascular parameter of remodeling cSBP was not different between two groups, also not after adjustment in models 1 and 2. No correlation was found between cPP and diabetic disease duration, FPG and HBA1c.

## Discussion

Microvascular complications in T2DM confer an excess risk of cardiovascular disease and death [27]. Microvascular and macrovascular complications often occur concomitantly after long-standing diabetes [28]. In this study we analyzed early clinical parameters in the renal, retinal and systemic circulation in a cross-sectional analysis and found three vascular parameters of organ damage, which can be clinically detected in T2DM patients with diabetic disease duration of median 4 years, thereby to be considered as early markers of diabetic disease progression (Table 2).

**Table 2:** Parameters in the micro- and macrocirculation

	Non-T2DM-subjects (n = 54)	T2DM-subjects (n = 111)	Unadjusted p-value	Adjusted p-value (model 1)	Adjusted p-value (model 2)
Retinal parameters					
ICD (µm)	20.8 ± 3.5	23.9 ± 5.1	< 0.001	< 0.001	0.001
RCF (AU)	310.4 ± 55.1	297.8 ± 72.9	0.15	0.35	0.72
WLR	0.35 ± 0.08	0.38 ± 0.11	0.04	0.67	0.90
Renal parameters					
eGFR (ml/min/1.73 m^2^)	95.9 ± 17.3	91.7 ± 9.9	0.10	< 0.001	< 0.001
UACR (mg/g)	7.9 ± 7.5	21.3 ± 86.6	< 0.001	0.55	0.91
Vascular stiffness parameters of large arteries (mmHg)					
cSBP	106.7 ± 12.4	119.5 ± 12.9	< 0.001	0.37	0.81
cPP	34.8 ± 10.6	41.8 ± 11.7	0.001	0.31	< 0.001

Data are given as mean ± SD

*ICD* intercapillary distance, *RCF* retinal capillary flow, *WLR* wall to lumen ratio, *eGFR* estimated glomerular filtration rate, *UACR* urinary albumin creatinine ratio, *cSBP* central systolic blood pressure, *cPP* central pulse pressure, *model 1* p-values are adjusted for age and gender, *model 2* p-values are adjusted for age, gender, *BMI*, fasting plasma glucose, *HbA1c* and serum HDL-cholesterol

Arterial stiffness is highly predictive of cardiovascular events [29]. One of the parameters, which we found was an increased cPP, which is a surrogate marker of arterial stiffness [30, 31], in patients with T2DM compared to patients without T2DM irrespective of BP of the study population. The mechanism of arterial stiffness in patients with early stage of T2DM, in which vascular calcifications are not yet expected, is not entirely understood. The association of BP with arterial stiffness due to mechanical stretch is well established [32]. However, ambulatory and office BP between the 2 groups in this study were not different and BP was well controlled. Hyperglycemia is known to cause oxidative stress, which induce endothelial dysfunction and damage of vascular function [33–35]. Our patients with T2DM had higher FPG compared to patients without T2DM. Hyperglycemia has been also shown to augment indices of arterial stiffness, especially in people with reduced insulin sensitivity and in patients with T2DM [36, 37]. The difference in cPP between our patients with and without T2DM remained significant even after adjustment with FPG and HbA1c. Thus, the glucotoxic effect does not entirely account to the observed difference in cPP between the two groups.

The improvement of arterial stiffness parameter pharmacologically has been repeatedly examined. Recently we could demonstrate that SGLT2-inhibitors are able to improve central hemodynamic parameters like cPP in patients with T2DM, which might be one of the reasons for the observed improved cardiovascular and renal outcomes with empagliflozin treatment in the EMPA-REG OUTCOME study [38, 39]. In accordance Chilton et al. determined significant reductions of cPP compared to placebo in a post hoc analysis of five phase III trials with T2DM patients being treated with empagliflozin [40].

Second vascular parameter, which we found to be different between the 2 groups, was increased ICD, which is a measure of capillary rarefaction. This finding of capillary rarefaction is in line with several previous studies [39, 41, 42]. Using macular optical coherence tomography, angiography images of the retinal capillary layers were obtained in 67 patients with T2DM. The fractal dimensional parameter, which quantifies the complexity and density of the two retinal capillary layers, was significantly lower in diabetic patients with no diabetic retinopathy, similar to our patients in early stage of T2DM, compared to controls [41]. In contrast, once diabetic retinopathy has developed and becomes clinically detectable fractal dimensional parameter increases [42]. Also, Simonett et al. could show that diabetic patients with no or mild signs of retinopathy have reduced parafoveal vessel density in the deep capillary plexus of the retina [43]. Signs of capillary rarefaction have been also shown in the nail fold capillaries of T2DM patients using nailfold videocapillaroscopy [44]. Our results indicate that SLDF measurements should be done early in patients with T2DM, thereby early indicating microvascular alterations in T2DM. Furthermore, retinal capillary rarefaction has been found to be improved after antihypertensive therapy [23]. We analyzed in parallel other retinal parameters (WLR, RCF), which are found to be impaired in hypertensive patients [45]. However, in comparison between our patients with and without T2DM, we did not find any difference of this parameter reflecting vascular remodeling in the retinal arterioles.

The third parameter which we found in our study population with early stage of T2DM was reduced eGFR compared to the subject without T2DM. All subjects in both groups had an eGFR above 60 ml/min/1.73 m^2^. We used the CKD-EPI formula to calculate eGFR, which has been found to be valid also in the upper normal range compared to the MDRD formula [24]. One confounder of eGFR is the volume and sodium status of the study population. However, eGFR was above 60 ml/min/1.73 m^2^, thereby decreasing the risk of having sodium and volume retention and since hematocrit was not different between the two groups, we have no signal that different volume status is a confounder for the difference in eGFR between the two groups. After adjustment with age and BP, the difference in eGFR remained significant. However, since eGFR was calculated, with the difference being small, eGFR might not be suitable as early marker of vascular disease.

In several studies the outcome of micro- and macrovascular complications in T2DM patients with intensive or standard therapy (retinopathy, nephropathy and cardiovascular events) have been reported [46–48]. Scarcely there have been reports comparing early vascular changes in the micro- and macrocirculation in the same patient population. Novel about our findings is that we assessed in parallel early vascular changes in different vascular beds in the same patient, thereby allowing us to compare vascular remodeling in the micro- and macrocirculation of patients in early stage of T2DM. Furthermore, we used advanced methods to assess early vascular remodeling. In contrast to other studies [49, 50], we do not use any mydriatic agents to dilate the pupil, which are known to influence the assessed retinal parameters [51].

## Limitations

Our study has several limitations. First, central hemodynamic parameters like cPP were non-invasively assessed, but the validity of the device used has been tested against invasive measurements and reproducibility has been shown in routine ambulatory settings [52, 53]. Second, our ICD measurements are based on perfusion images and our method, similar to the method of assessing capillary rarefaction in the skin [54], does not allow to differentiate between functional and structural vessel changes. However, the reliability of this method has been found to be acceptable [23]. Third, renal function in our population was estimated using the CKD-EPI formula and not measured by inulin clearance, but it has been shown that estimated GFR is accurate, precise and least biased [24, 55, 56]. Furthermore, our results are only valid for patients with T2DM with median diabetes duration of 48 months and cannot be extrapolated to other stages of T2DM or type-1 diabetes. The clinical characteristics of subjects with T2DM, especially age and gender, differ significantly compared to subjects without T2DM, so that our results have been adjusted using covariance analyses for these characteristics, but it should be noted that statistical adjustment has its own limitations. Finally this study is a cross-sectional study, so we cannot be certain that these vascular changes at early stage of diabetes are the earliest in the course of diabetic disease. Further longitudinal examination of these abnormalities is needed.

## Conclusion

Our data suggest that at similar blood pressure capillary rarefaction in the retinal circulation, decreased eGFR in the renal circulation and increased central pulse pressure of large arteries are early detectable in patients with T2DM compared to non-T2DM subjects. Therapeutic interventions should address these early vascular changes appropriately.

## Abbreviations

T2DM: type 2 diabetes mellitus
FBG: fasting plasma glucose
WLR: wall-to-lumen ratio
RCF: retinal capillary flow
ICD: inter capillary distance
cSBP: central systolic pressure
cPP: central pulse pressure
UACR: urinary albumin creatinine ratio
